# Non-Invasive Measurement of Elasticity in Glioblastoma Multiforme Validates Decreased TMZ Sensitivity in Astrocyte Co-Culture

**DOI:** 10.1109/OJEMB.2025.3528194

**Published:** 2025-01-10

**Authors:** Megan Mendieta, Maryam Hatami, Manmohan Singh, Sajedeh Saeidi Fard, Mohammad Dehshiri, Alexander Schill, Dmitry Nevozhay, Salavat Aglyamov, Bulent Ozpolat, Konstantin V. Sokolov, Yasemin M. Akay, Kirill V. Larin, Metin Akay

**Affiliations:** University of Houston 14743 Houston TX 77204 USA; University of Texas MD Anderson Cancer Center 4002 Houston TX 77030 USA; Houston Methodist Research Institute 167626 Houston TX 77030 USA; University of Texas MD Anderson Cancer Center 4002 Houston TX 77030 USA; Rice University 3990 Houston TX 77030 USA

**Keywords:** Brillouin, GBM, OCE, spheroid, stiffness

## Abstract

*Goal:* In this research, we investigated the changes in elasticity of in vitro glioblastoma multiforme (GBM) spheroids when treated with the gold standard chemotherapy for GBM, Temozolomide (TMZ). Additionally, we aimed to use this alternative biomarker to assess how modifying the tumor microenvironment (TME) with the addition of human astrocytes (HA) would influence treatment efficacy. *Methods:* Spheroid stiffness was investigated using advanced non-invasive optical techniques, nanobomb optical coherence elastography (nb-OCE) and Brillouin microscopy to obtain new biomechanical insights by assessing local tumor progression or response to therapy using GBM cells (LN229). *Results:* The treated monocultured GBM groups showed a significant decrease in stiffness and increased sensitivity to treatment with TMZ. Treated HA groups across approaches remained relatively unchanged in stiffness. Treated co-culture groups demonstrated significant resistance to treatment with TMZ, where stiffness decreased less than that of the treated LN229 cells. *Conclusions:* These results confirm earlier findings using cell viability as a biomarker for treatment efficacy, making nb-OCE and Brillouin promising options to probe 3D tumor models in vitro non-invasively.

## I. Introduction

Glioblastoma multiforme (GBM) is a grade IV astrocytoma, representing 90% of the cases of brain tumors. It is a highly aggressive and heterogeneous subtype that arises from glial cells, which are non-neuronal cells that provide metabolic, homeostatic, and structural support to protect neurons in the central nervous system [1], [2]. Over 90% of GBM cases develop from normal glial cells through multistep oncogenesis, which involves genetic aberrations and mutations leading to the activation of multiple signaling pathways [3], [4]. Management of GBM includes surgical tumor resection, chemotherapy, where the gold standard is Temozolomide (TMZ), and radiotherapy [5]. The median overall survival rate of patients diagnosed with GBM is 14 months with treatment and 3-4 months without treatment [6]. The five-year survival rate is only 6.8%, seemingly the lowest among all brain tumor types, where long-term survival of 10 years or more makes up less than 1% of cases [7].

Tracking patient response to treatment is challenging for most cancers in general, but it is especially difficult in GBM due to a high degree of heterogeneity that may not be revealed even in a biopsy [8]. Imaging of GBM, commonly during diagnosis and evaluation of therapeutic response, employs invasive procedures such as catheter angiography and non-invasive methods such as computed tomography (CT) and, most frequently, magnetic resonance imaging (MRI), which has superior soft tissue contrast to better determine tumor complexity and heterogeneity [9]. Current imaging techniques often underestimate tumor invasion, making it difficult to assess progression [10], [11]. In order to assess treatment efficacy, in vitro techniques often include cell viability experiments; this is an end-point analysis, and identification of multiple biomarkers would yield a better perspective on the progression of the tumor.

Understanding the biomechanical properties of tumors is crucial because such properties can affect response to therapies, influence tumor progression via cell cycle regulation and migration, and contribute to therapeutic resistance [12]. The need for further research and the development of advanced imaging techniques is evident, particularly where elasticity may serve as an effective biomarker [13], [14], [15], [16], [17].

Optical coherence elastography (OCE) and Brillouin microscopy are two non-invasive and high-resolution biomechanical imaging techniques. Wave-based OCE offers a new exciting way to assess tissue mechanical properties completely non-invasively. It benefits from nanometer scale displacement sensitivity by using phase-sensitive optical coherence tomography (OCT) to detect the tissue deformation response after an excitation to form elastograms [18], [19]. Brillouin microscopy investigates the mechanical properties of the material through photon-phonon interactions, so it does not need to deform the sample with an external load and can achieve sub-micron spatial resolution using a high numerical-aperture (NA) objective lens [20], [21]. These properties make wave-based OCE and Brillouin microscopy suitable for quantitative and local assessments of viscoelasticity changes during, e.g., organoid and embryonic development [22] and tumor treatment [23].

The GBM tumor microenvironment (TME) contains not only tumor cells but also diverse cell types, including astrocytes. Astrocytes make up about 50% of all brain cells, facilitate inter-cell communication, provide structural support, and are associated with creating and maintaining neural circuits [24]. Tumor-associated astrocytes can promote cell proliferation and migration, as well as contribute to neuroinflammatory responses leading to tumor growth and progression [25]. In our previous work, primary human astrocytes were co-cultured with the LN229 GBM cell line in 3D, demonstrating increased GBM survival and resistance after combined TMZ treatment compared to monocultures [26]. However, there is a lack of comprehensive studies on the biomechanics of the pharmacologically induced, temporal changes in brain cancer spheroids. Such studies may be used to further validate the link between tumor necrosis and decreasing tumor stiffness, as well as to probe the effects of different cell-to-cell interactions [27]. So, in this study, we aimed to understand if treatment with the first-line standard chemotherapy drug, TMZ, induces a change in brain cancer spheroid elasticity, depending on the types of cells present, as measured by OCE and Brillouin microscopy. In agreement with our previous work, when LN229 is monocultured, we predicted spheroid necrosis and a correlated decrease in stiffness, greater than the necrosis and decrease in stiffness observed when co-cultured with human astrocytes [26].

## II. Materials and Methods

### A. Cell Lines and Culture

Glioblastoma cell line LN229 was purchased from the American Tissue Culture Collection (ATCC, USA). LN229 cells were cultured in a cell culture plate up to passage 11, using Dulbecco's modified Eagle's medium (DMEM) (Gibco, USA) supplemented with 10% FBS (Gibco, USA), and 1% of 100 U/mL penicillin and 0.1 g/mL streptomycin (Gibco, USA). A primary Human Astrocyte (HA) cell line from the cerebral cortex was purchased from Sciencell (USA) and cultured up to passage 6, using Poly-L-Lysine coated flasks, in specialty Astrocyte Medium (Sciencell, USA), 2% FBS (Sciencell, USA), 1% Astrocyte Growth Serum (Sciencell, USA), and 1% of 10000 units/mL of Penicillin and 10000 μg/mL of Streptomycin in a saline solution (Sciencell, USA). All cells were stored in a cell culture incubator at 5% CO_2_, 37 °C.

### B. Microwell Fabrication and Cell Seeding

Poly(ethylene glycol) diacrylate (PEGDA) microwells on a chip were generated as described previously [28]. Cover glass slides (24 × 24 mm^2^) were treated with 3-(Trimethoxysilyl) propyl methacrylate 98% (TMSPMA) (Life Technologies, USA). The slides were first layered with a 20 μL of 40% (w/w) PEGDA (MW 700) (Life Technologies, USA), 0.2% (w/v) photoinitiator (PI) 2-hydroxy-2-methyl propiophenone, and Phosphate Buffered Saline (PBS) (Life Technologies, New York, NY, USA) solution. They were then exposed to Lumen Dynamics the OmniCure Series 2000 (Lumen Dynamics Group Inc, Canada) for 36 seconds at a working distance of 6 inches. Next, 250 μL of PEGDA solution was added to the slide and cured in UV light for 36 seconds with a photomask (CADart, USA) with 1000 μm diameter dots in a grid pattern, on top. Prepared slides were washed and incubated overnight in 6-well plates containing 2 mL of PBS each.

The PBS was removed, and cells were seeded at a concentration of 0.4 × 10^6^ cells/mL on each microwell in cell culture media droplets of 190 μL, which were allowed to incubate at room temperature for 5 minutes. The remaining 1810 μL of cell culture media was added for a final volume of 2 mL per well. LN299 spheroids were cultured in DMEM based media. HA spheroids and co-cultured LN229 and HA spheroids (1:1 seeding ratio) were cultured in specialty Human Astrocyte based media. The plates were placed in the cell culture incubator and 1 mL of warmed cell culture media was exchanged every 2 days for a week to allow for spheroid aggregation. Spheroid formation was monitored using a microscope (Olympus, Japan).

### C. Drug Treatment of LN229, HA, and Co-Culture Spheroids

The gold standard chemotherapy treatment, TMZ, was applied to the spheroids by first removing the existing cell culture media and diluting TMZ to a working concentration of 300 μM in 2 mL of appropriate cell culture media. The solution was then gently administered to the side of the wells and the spheroids were placed back into the incubator to be monitored over the course of a week using a brightfield microscope (Olympus, Japan). Control untreated microwells versus treated microwells were also assessed using nb-OCE, Brillouin microscopy, and OCT imaging.

### D. Preparation and Characterization of Nanoparticles

The preparation of dye-loaded nanodroplets, termed nanobombs, followed a modified version of a previously described protocol [29], [30], [31], [32]. The process involved combining 18 mg 12-distearoyl-sn-glycero-3-phosphocholine (DSPC), 1.36 mg 12-distearoyl-sn-glycero-3-phosphoethanolamine-N-methoxy-polyethyleneglycol-2000 (DSPE-PEG-2000), 0.36 mg 12-distearoyl-sn-glycero-3-phosphoethanolamine-N-biotinyl-polyethyleneglycol-2000 (DSPE-PEG-2000-Biotin), 0.3 mg cholesterol (all sourced from Avanti Polar Lipids, Inc., USA), and 1 mg Epolight 3072 dye (Epolin Inc., USA) in 2 mL chloroform. The lipid-dye mixture in chloroform was heated to 40 °C using a rotary evaporator (Cole-Parmer Instrument Company, LLC, USA) and vacuum-dried for 20 minutes to create a thin lipid film. The film was rehydrated with 2 mL deionized water and agitated for 30 minutes at 250 rpm. Next, 150 μL of tetradecafluorohexane (Sigma-Aldrich Inc., USA) was introduced to the hydrated lipids, followed by 20 seconds of vortexing and 30 seconds of sonication in an ice-cold water bath using a benchtop ultrasonic cleaner (CPX-962-218R, Fisher Scientific, USA). The mixture was then vortexed for 30 seconds again, followed by two 1-minute cycles of probe sonication at 25% maximum amplitude using a VCX 500 ultrasound probe (Cole-Parmer Instrument Company, LLC, USA), interspersed with 20 seconds of vortexing. The resulting suspension was washed three times with 2 mL deionized water, centrifuged at 3100 × g for 15 minutes, and the final pellet was resuspended in 1 mL deionized water. Nanodroplet size was determined using dynamic light scattering (Malvern Zetasizer Pro, Malvern Panalytical Ltd., U.K.). The washed nanodroplets measured 216 ± 9 nm in size, with a PDI of 0.158. Perfluorocarbon concentration was quantified via ^19^F Nuclear Magnetic Resonance (NMR). For NMR analysis, 10 μL of sample was mixed with 75 μL of 0.5% trifluoroacetic acid in deuterium oxide (both from Sigma-Aldrich Inc., USA) and 400 μL of deionized water. Measurements were conducted in 5 mm NMR tubes (Wilmad-LabGlass, USA) using a 500 MHz NMR spectrometer (Bruker Corporation, USA) with the following parameters: 241.5 ppm spectrum width, -80 ppm central frequency, 1 dummy scan, 0.58 s acquisition time, 16 signal averages (with phase cycling), and 15 s relaxation delay. The ^19^F concentration in the preparation (3.17 M) was calculated by integrating the peaks and normalizing them to a known concentration of trifluoroacetic acid standard. Prior to cellular application, nanodroplets were diluted to 100 mM ^19^F concentration in the same media used for spheroid culture.

### E. Optical Coherence Tomography (OCT) and Sample Preparation

The diagram of the OCT setup can be found in Supplemental Fig. 1. The phase-sensitive, spectral domain OCT system included a superluminescent diode (SLD, S840-B-I-20, Superlum Co., Ireland) with a central wavelength of 840 nm and bandwidth of 49 nm. The spectral interference was detected by a spectrometer (CS800-840/120-250-OC2K-CL, Wasatch Photonics, USA), and the camera rate was set to 40 kHz. The axial and lateral resolutions of the system were measured as 5.4 μm and 9.8 μm in air, respectively. The scan area captured 0.8 mm^2^ and 1000 A-lines per B-scan.

The microwell chip was covered with 200 μL of unsupplemented DMEM, which was reapplied as needed to keep the gel hydrated. Three-dimensional OCT imaging was performed on the co-culture spheroids at days 0, 4, and 7 to visualize changes to composition over time. The OCT images were acquired with 1000 A-lines per B-scan and 1000 B-scans per volume, covering a volume of 0.8 mm × 0.8 mm × 0.3 mm refers to X, Y, and Z respectively. Each B-scan was repeated three times to average the data in post-processing and enhance the signal-to-noise ratio (SNR). Images were then analyzed and reconstructed to create 3D models of the spheroids

### F. Nanobomb-Optical Coherence Elastography and Sample Preparation

The specifications of the nb-OCE setup can be found in Supplemental Fig. 2. The 8× buffered Fourier domain mode-locked (FDML) swept source laser-based OCT system (OMESv2, OptoRes GmbH, Germany) had an A-scan rate of 3.2 MHz, central wavelength of 1310 nm, and a wavelength scan range of 100 nm. Axial resolution was 10 μm, sensitivity was 100 dB, displacement stability was 2.6 nm (13 nm with buffering), and the power on the sample was 33 mW. Data acquisition was performed at 4 GS/s (ATS9373, AlazarTech, Canada).

The nanobomb solution was prepared for same-day use, as described [29], [33]. A volume of 200 μL of solution was initially applied as a droplet atop the prepared PEDGA microwells containing control or treated spheroids. The solution was allowed to incubate and settle into microwells for 5 minutes before another 200 μL was applied. After 5 minutes more of incubation, the wetted microwell chip was placed under the apparatus, where a spheroid was visualized and centered under the pulsed laser, as shown in Supplemental Fig. 3. The nano particles surrounding the spheroid were then excited by the Q-switched Nd:YAG laser (Polaris II, New Wave Research, Inc., USA) with a single 6 ns pulse duration to induce mechanical waves within the spheroid. The pulsed laser wavelength was set at 1064 nm. The laser beam diameter was 1.2 mm, and measurements were performed at ∼0.75 mJ laser energy providing the laser fluence of 120 mJ/cm^2^. With the nb-OCE setup, the longitudinal component of the shear wave was imaged [29]. The nanobombs were repeatedly activated to collect wave velocity measurements via the slope of the shear wave propagation delays vs. axial distances in the depth-time map in triplicate [31]. The elasticity was then calculated from the wave speed based on the shear wave equation [18], [29]. Measurements were collected on days 0, 4, and 7 to analyze mechanical changes to the spheroids over time.

### G. Brillouin Microscopy and Sample Preparation

The specifications of the Brillouin setup can be found in Supplemental Fig. 4. The Brillouin microscopy system used a 660 nm single longitudinal mode laser source (Torus, Laser Quantum Inc., CA) and a two-stage virtually imaged phased array (VIPA) spectrometer, which captured backscattered light from the sample. The backscattered light was relayed to the dual VIPA spectrometer, and the Brillouin frequency shift of the sample was detected by an electron-multiplying charge-coupled device (EMCCD) camera (iXon Andor, U.K.). The system was calibrated with water, acetone, and methanol before every measurement. The incident power on the sample was 17 mW, the free spectral range was ∼30 GHz, and the camera acquisition time was 0.1 s per spectrum. The sample was imaged with a 4× objective with a 0.8 numerical aperture (NA), resulting in an axial resolution of 2.3 μm and a lateral resolution of 1.8 μm as measured using a beam profiler (LaserCam-HR II, Coherent Inc., USA). An integration time of 0.2 s was used during all measurements.

The microwell chip containing control or treated spheroids was placed on a 26 × 76 mm microscope slide for stability, covered with a 200 μL unsupplemented DMEM droplet, and then covered with a 24 × 24 mm glass cover glass. The construct was then placed on the sample holder and the frequency change of scattered light was analyzed to quantify the Brillouin shift, which was collected in triplicate (Supplemental Fig. 5). As with OCE, the Brillouin frequency shift was analyzed over time, at days 0, 4, and 7.

### H. Statistical Analysis

All reported results were from three independent experiments performed in triplicate. The Kruskal-Wallis test and Mann-Whitney U test for non-parametric data were utilized for statistical comparisons between experimental groups and longitudinal samples, where a p-value < 0.05 indicated a statistically significant difference between values. The data were displayed as the average ± standard deviation.

## III. Results

### A. Optical Coherence Tomography

**Fig. 1.**
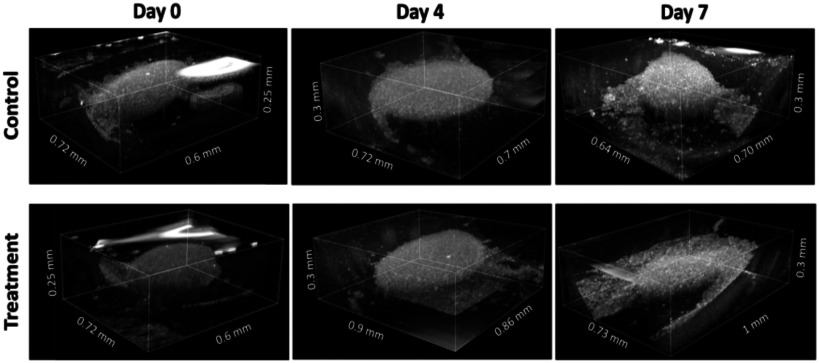
3D OCT imaging of co-cultured spheroids. Untreated versus TMZ-treated LN229 and HA spheroids imaged using an SD-OCT system (Supp Fig. 1). For the treated group, chemotherapeutic agent TMZ was applied at day 0 and all spheroids were imaged over 7 days.

To observe the changes in 3D structure of the co-culture spheroids over the experimental time period, 3D OCT imaging was performed. The microwell platform was prepared as mentioned in Methods. High-resolution, cross-sectional images were stacked to generate a 3D rendering of an individual spheroid within a microwell. The untreated co-cultured spheroids maintained their ovoid shape over the 7 days, whereas the treated spheroids began to come unbound and misshapen, which is characteristic of cellular necrosis as represented in the nb-OCE and Brillouin experimental results (Fig. 1).

### B. Nanobomb-Optical Coherence Elastrography

To investigate the changes in elasticity of monocultured LN229 spheroids, as well as monocultured HA spheroids and co-cultured LN229 and HA spheroids, nb-OCE was first employed. The microwell platform was prepared as mentioned in Methods. An individual spheroid within the microwell was aligned within the system and imaged using a modular inverted microscope setup below the microwell dish. The microscope consisted of a 2.5× objective lens and CMOS sensor (LaserCam-HR-II, Coherent, USA). Upon pulsing of the laser, we were able to determine the state of nanobomb activation (Supplemental Fig. 6). Data could only be collected if the spheroids were properly surrounded by nanobombs via deposition of the nanobomb solution down into the microwells. The slope of the time delays vs. depth was utilized to determine the speed of the nanobomb-induced longitudinal shear wave, propagating through the spheroid, as shown in Supplemental Fig. 6(b) [32]. The speed of the wave was then used to estimate Young's modulus by using the shear wave equation with Poisson's ratio of 0.49 and assuming 1000 kg/m^3^ for the density of samples. Two-dimensional images of each spheroid being probed were also collected to monitor their changes in size, confirming the replication of our previous work [26].

**Fig. 2.**
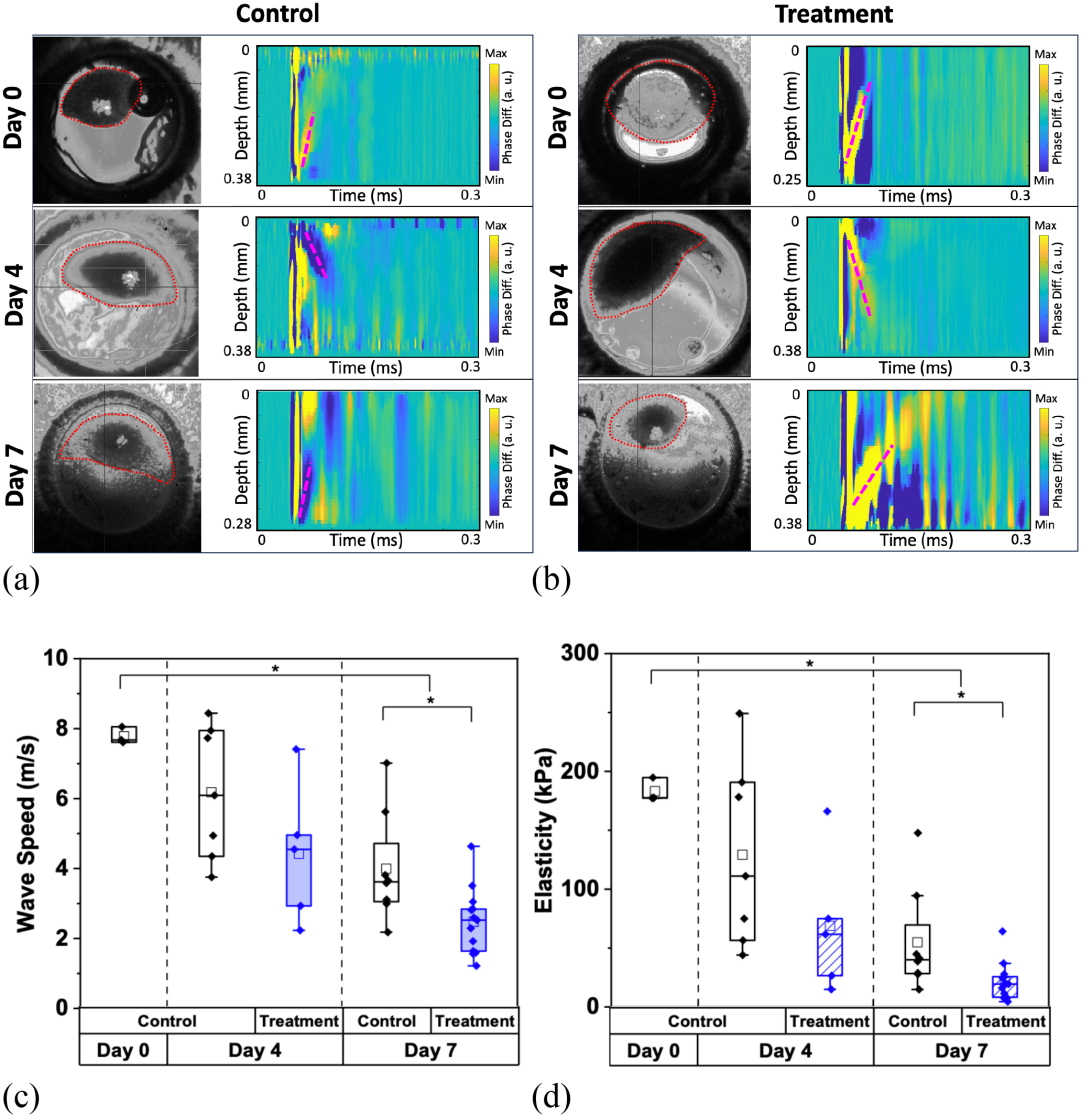
Nb-OCE wave speed and elasticity of LN229 spheroids. (a) Untreated monocultured LN229 spheroids and (b) TMZ-treated spheroids images over the course of 7 days, paired with their spatiotemporal maps and (red dashed line) wave propagations following nanobomb activation. (c) Comparative wave speeds of control and treated LN229 spheroids over a 7-day period and their (d) correlated elasticity values. For the treated group, chemotherapeutic agent TMZ was applied at day 0, and all spheroids were probed on days 0, 4, and 7. Data represents the mean ± SD of three biological replicates.

As discussed in the methods, half of the LN229, HA, and co-culture spheroids were administered TMZ at 300 μM concentration. The TMZ-treated LN229 spheroids demonstrated a significant decrease in stiffness (p = 0.0003 by Kruskal-Wallis ANOVA for both control and treated groups), as plotted in Fig. 2(c) and (d), over the 7-day period, where the control (i.e., untreated) spheroids wave speed decreased at a much slower rate. However, the stiffness for the treated spheroids was significantly less than the stiffness of the control samples (p = 0.01 by U-test). It was also observed that the size of the spheroids in the treated wells decreased, while the control spheroids increased in size, as shown in Fig. 2(a) and (b), highlighting changes in cell viability that agree with our previous work [26].

**Fig. 3.**
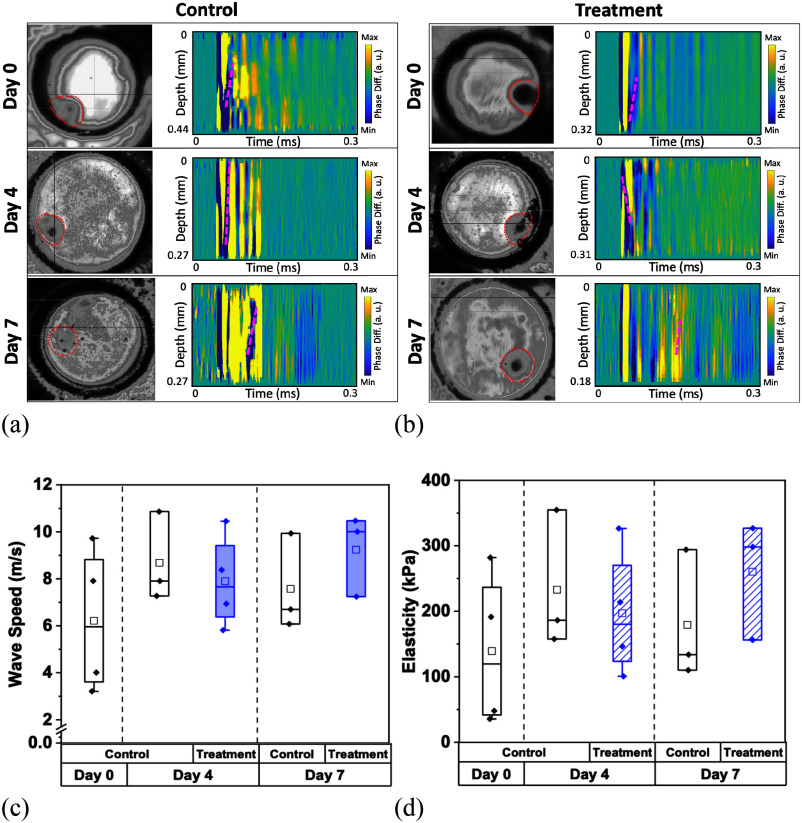
Nb-OCE wave speed and elasticity of HA spheroids. (a) Untreated monocultured HA spheroids and (b) TMZ-treated spheroids images over the course of 7 days, paired with their spatiotemporal maps and (red dashed line) wave propagations following nanobomb activation. (c) Comparative wave speeds of control and treated HA spheroids over a 7-day period and their (d) correlated elasticity values. For the treated group, chemotherapeutic agent TMZ was applied at day 0, and all spheroids were probed on days 0, 4, and 7. Data represents the mean ± SD of three biological replicates.

The TMZ-treated and untreated control HA spheroids maintained a similar stiffness (p > 0.05 by Kruskal-Wallis ANOVA for both control and treated groups), over the 7-day period, as illustrated in Fig. 3(c) and (d). Relatedly, the spheroid size of both experimental groups decreased slightly but remained relatively similar over time (p > 0.05 by U-test), as shown in Fig. 3(a) and (b); this is also in agreement with the cell viability findings of our previous study [26].

**Fig. 4.**
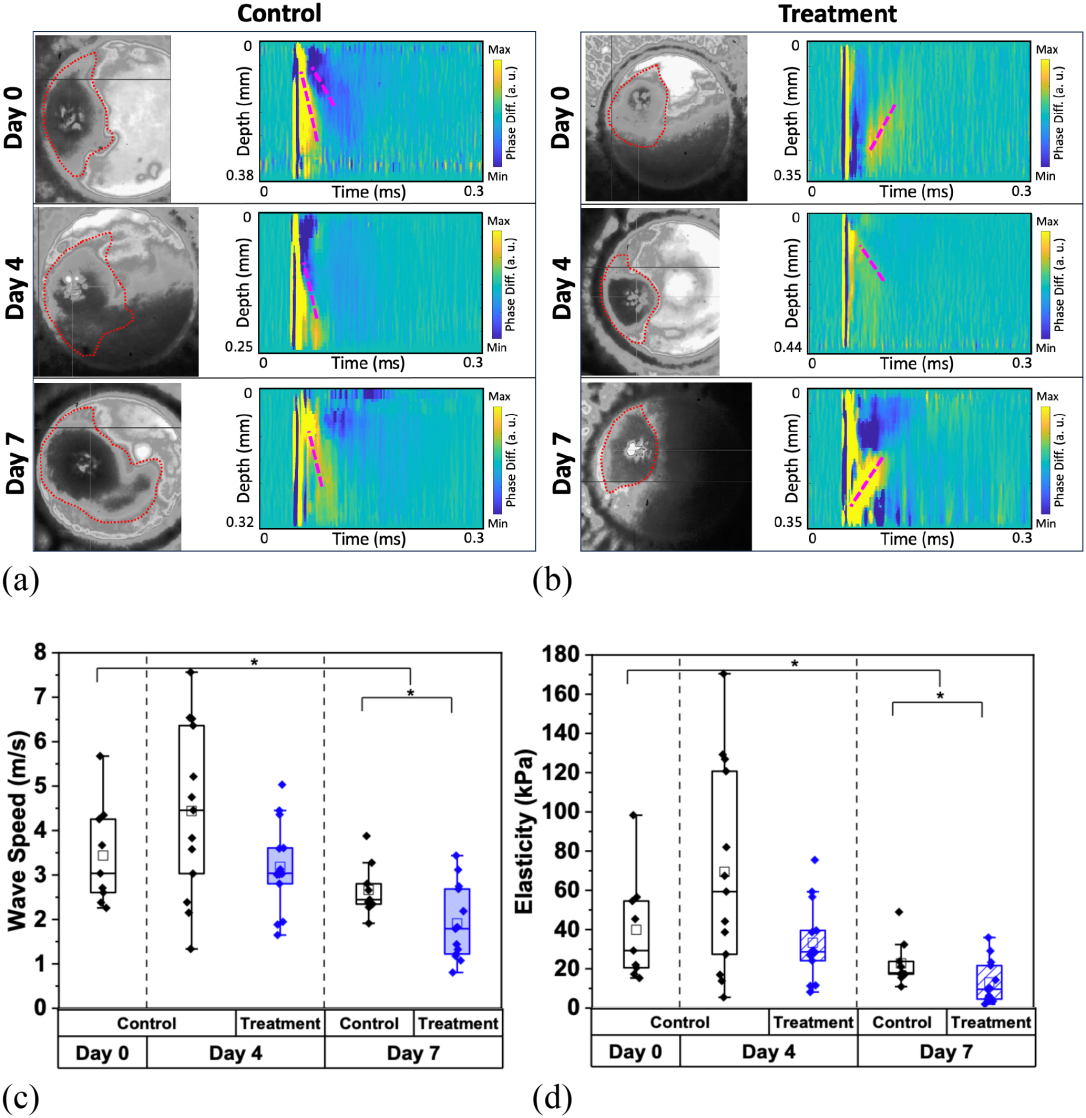
Nb-OCE wave speed and elasticity of LN229 and HA co-cultured spheroids. (a) Untreated co-cultured LN229 and HA spheroids and (b) TMZ-treated spheroids images over the course of 7 days, paired with their spatiotemporal maps and (red dashed line) wave propagations following nanobomb activation. (c) Comparative wave speeds of control and treated co-cultured spheroids over a 7-day period and their (d) correlated elasticity values. For the treated group, chemotherapeutic agent TMZ was applied at day 0, and all spheroids were probed on days 0, 4, and 7. Data represents the mean ± SD of three biological replicates.

Lastly, when observing the co-cultured LN229 and HA spheroids, they seemed to display resistance to the TMZ treatment because its effect resulted in a reduced decrease of the spheroids’ stiffness over time (p = 0.0008 by Kruskal-Wallis ANOVA for both control and treated groups) as plotted in Fig. 4(c) and (d) compared to Fig. 2(c) and (d). The size of the treated spheroids, shown in Fig. 4(a) and (b), only slightly decreased (p = 0.04 by U-test), and certainly less than that of the LN229 monoculture spheroids; this too aligns with the results of the previous studies.

### C. Brillouin Microscopy

Brillouin microscopy was utilized as a secondary means of characterization of the mechanical properties of the monocultured LN229 spheroids, monocultured HA spheroids, and co-cultured LN229 and HA spheroids. The microwell platform was prepared as mentioned in Methods. Each individual spheroid was aligned within the system, and the frequency shift of the backscattered light was analyzed for a single representative central plane in the Z-axis of the spheroid, i.e., an axial slice. The treatment groups of LN229, HA, and co-culture spheroids were administered TMZ at 300 μM concentration on day 0 of the experiment and monitored for 7 days; untreated groups received only supplemented DMEM.

**Fig. 5.**
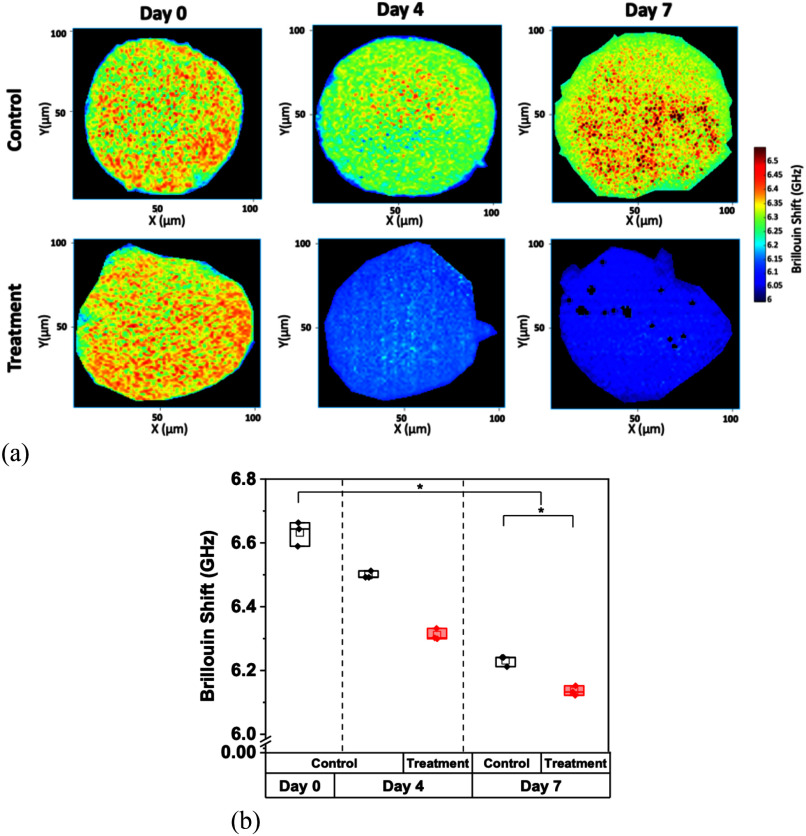
Brillouin shift measurements of LN229 spheroids. (a) Representative Brillouin shift images over the course of 7 days of untreated monocultured LN229 spheroids and TMZ-treated spheroids. (b) Comparative average Brillouin shift measurements of control and treated LN229 spheroids over 7 days. For the treated group, the chemotherapeutic agent TMZ was applied at days 0, and all spheroids were imaged on day 0, 4, and 7. Data represents the mean ± SD of three biological replicates.

The LN229 spheroids demonstrated a significant decrease in Brillouin shift values and correlated stiffness over the course of the experiment, as illustrated in Fig. 5 (p = 0.009, for the control and TMZ-treated spheroids, by Kruskal-Wallis ANOVA). Treated LN229 spheroids were significantly less stiff than their control counterparts at day 7 (p = 0.05 by U-test).

**Fig. 6.**
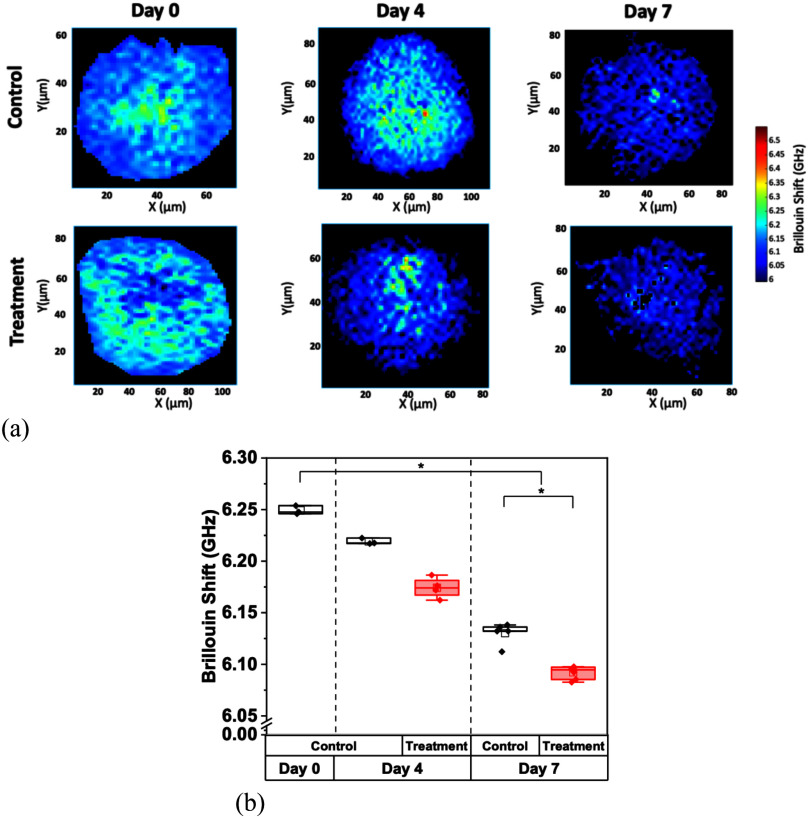
Brillouin shift measurements of HA spheroids. (a) Representative Brillouin shift images over the course of 7 days of untreated monocultured HA spheroid microwells and TMZ-treated spheroid microwells. (b) Comparative average Brillouin shift measurements of control and treated HA spheroids over 7 days. For the treated group, the chemotherapeutic agent TMZ was applied at days 0, and all spheroids were imaged on day 0, 4, and 7. Data represents the mean ± SD of three biological replicates.

Treated and untreated HA spheroids followed a similar pattern in showing a slight decrease in Brillouin shift values and stiffness (p = 0.002, for the control and TMZ-treated spheroids, by Kruskal-Wallis ANOVA) during the 7-day period (Fig. 6), with a significant difference between control and treated groups at day 7 (p = 0.02 by U-test).

**Fig. 7.**
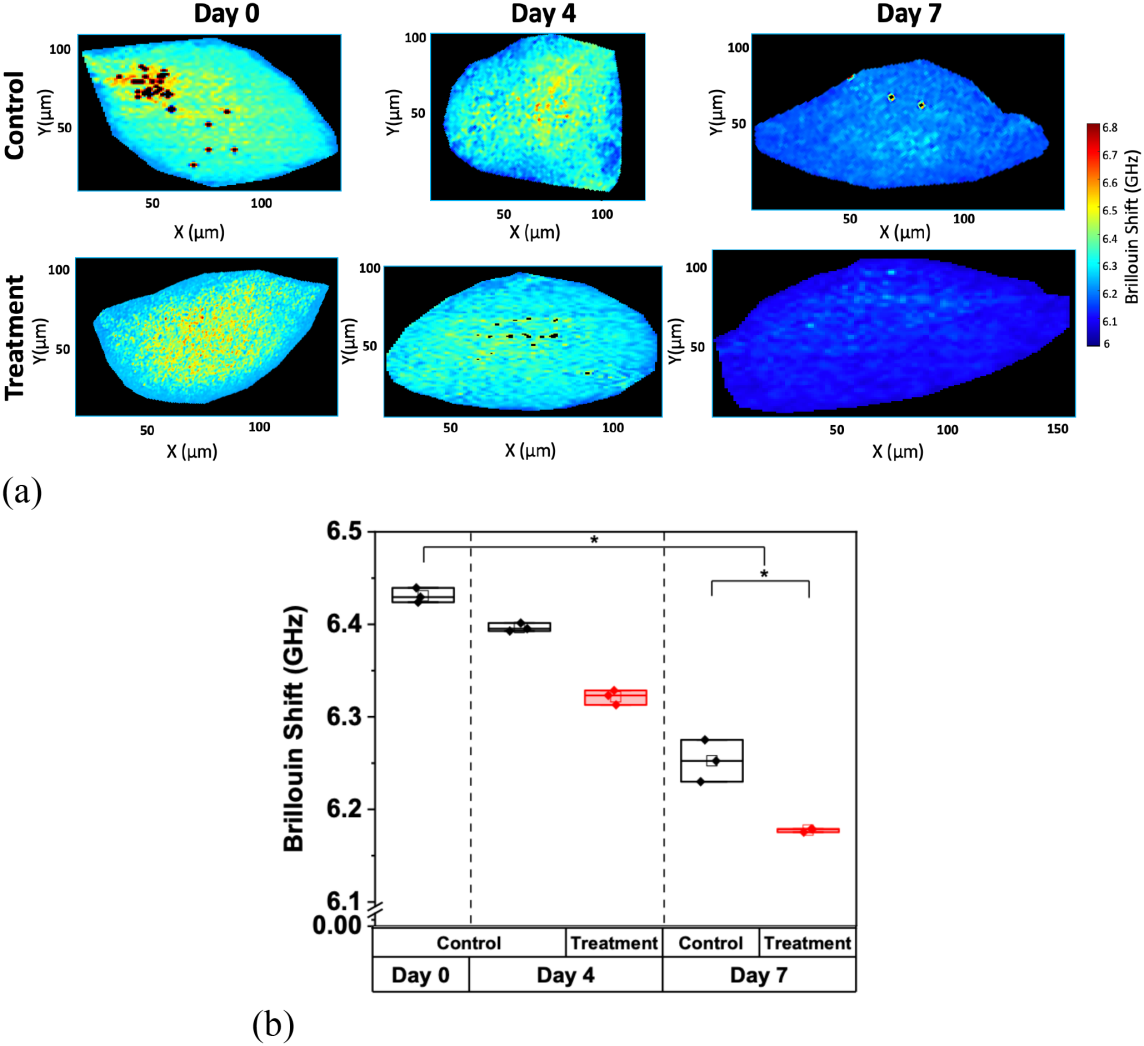
Brillouin shift of LN229 and HA co-cultured spheroids. (a) Representative Brillouin shift images over the course of 7 days of untreated co-cultured LN229 and HA spheroids and TMZ-treated spheroids. (b) Comparative average Brillouin shift measurements of control and treated co-cultured spheroids over 7 days. For the treated group, chemotherapeutic agent TMZ was applied at day 0, and all spheroids were probed on days 0, 4, and 7. Data represents the mean ± SD of three biological replicates.

Co-cultured spheroids showed a significant decrease in Brillouin shift values and stiffness over the course of the study (p = 0.009, for the control and TMZ-treated spheroids, by Kruskal-Wallis ANOVA). When treated with TMZ, the co-cultured spheroids showed a small decrease in Brillouin shift and, therefore, stiffness, versus the control group (p = 0.05, by U-test) by day 7 (Fig. 7). In comparison with the LN229 monoculture, the co-culture experimental group demonstrated a smaller Brillouin shift change by day 7 (p = 0.03, by U-test), indicating induced resistance to chemotherapeutic treatment via TMZ. These findings agree with our previous study, where a visual decrease in stiffness was correlated with an increase in treatability and a decrease in cell viability [26].

### D. Summary of nb-OCE Versus Brillouin Shift Findings

Nb-OCE and Brillouin microscopy are both non-invasive techniques that can assess the biomechanical properties of tumor-like tissues. Nb-OCE is faster and can quantitatively provide tissue shear elasticity, while Brillouin microscopy measurements are related to the bulk elastic modulus, but are slow [34].

**Fig. 8.**
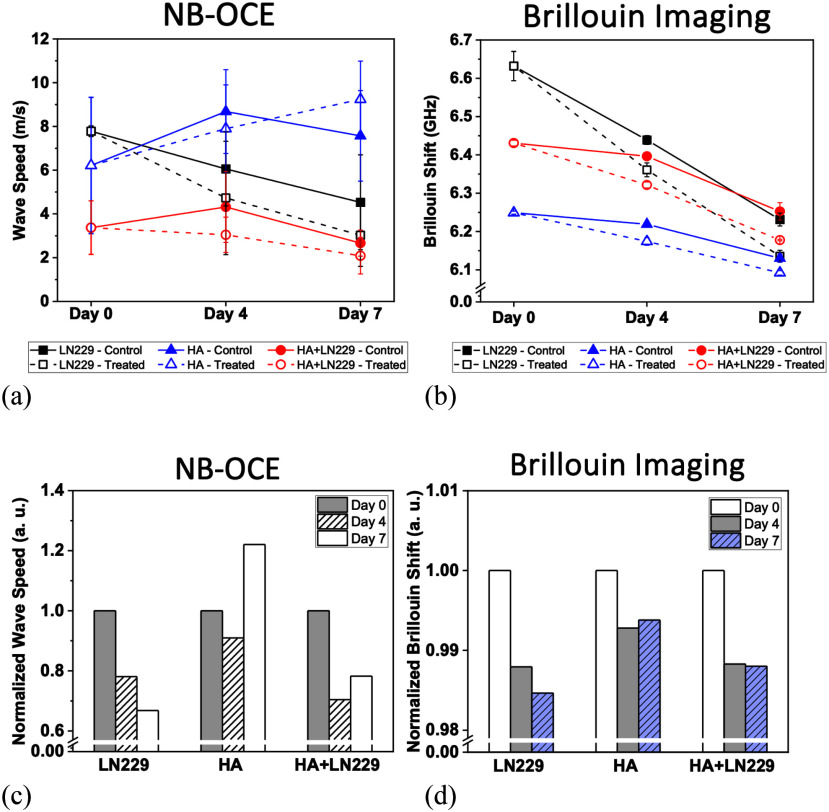
Summary of treatment effect on mono- and co-cultured spheroids. (a) Graph of the comparative shear wave speeds of the LN229, HA, and Co-Cultured spheroids over 7 days and the parallel (b) Brillouin shift measurements. (c) Graphs of the normalized results from nb-OCE experiments and (d) normalized results from the Brillouin microscopy. For the treated group, the chemotherapeutic agent TMZ was applied at days 0, and all spheroids were probed on days 0, 4, and 7. Data represents the mean ± SD of three biological replicates.

The two approaches can be compared and relatively demonstrate the differences in outcome, although it can be clarified further through normalization (Fig. 8). Normalization values were calculated as the ratio of treated to control values for shear wave velocity and Brillouin shift. It is understandable that each set of spheroids may have a differing baseline of stiffness at Day 0, but the important takeaway is the change in stiffness from each individual baseline stiffness and the change derived from the control to the treatment group. If observed in this manner, it can be understood that while the HA groups across approaches remain relatively unchanged in stiffness from control to treatment group, the LN229 treated groups show significant sensitivity to treatment with TMZ, and co-culture treated groups demonstrate significant resistance to treatment with TMZ.

## IV. Discussion

Nb-OCE has been utilized in earlier studies on multilayered tissue-mimicking phantoms to demonstrate discrimination of elasticity gradients along depth [32]. Applying this technique to the individual glioblastoma spheroids in our microwell platform, we were able to efficiently determine the wave speed and calculate elasticity values with high reproducibility. The concentration of the NBs was controlled to minimize the generation of multiple waves, and we were able to reduce further elements of noise with our initial setup for the experiment [32]. This led to clear visualization of the longitudinal shear waves, which were so distinct that they could be linked to specific depths or locations within the spheroid. These results show one of the first applications of dynamic OCE for measuring the stiffness of tumor spheroids as quasi-static OCE has been utilized for spheroid elasticity characterization but required physical contact with the samples, which may not be viable for longitudinal imaging [35]. The dynamic non-contact approach utilized here eliminates the need for contact, which could enable high-throughput longitudinal imaging.

Brillouin microscopy has been used recently to map the longitudinal elastic modulus with micro-scale resolution in tissues such as the crystalline lens or murine embryo neural tubes [21], [22]. Brillouin light scattering measures the frequency shift of photons that have interacted with acoustic phonons, where a larger shift in frequency generally indicates a stiffer substance, and a lesser shift in frequency indicates a more compliant substance [36]. This principle was applied to capture the mechanical properties of individual spheroids in our microwell platform. Although Brillouin microscopy is typically employed in clear substances, it is possible for the spheroids to be imaged due to their shallow ∼200 μm radius; beyond this, the spheroid may begin to become opaquer [37], limiting Brillouin microscopy. Brillouin microscopy, while lengthier in acquisition time (tens of minutes versus tens of milliseconds for nb-OCE), has a much finer spatial resolution (∼2 μm in this case) where specific areas of the spheroids could be studied for their differences in stiffness.

Previously, with PEGDA microwells, we have been able to study the effects of different chemotherapeutic drugs in the assay and the effect of human umbilical vein endothelial cells (HUVEC) on drug resistance using cell viability experiments. Employing new imaging biomarkers would aid in explaining tumor mechanisms and deliver supplementary data to help distinguish other key hallmarks of tumor pathophysiology before, during, and following a drug response [38], [39]. In this study, we alternatively investigated the changes in stiffness of in vitro GBM spheroids, when treated with TMZ, to monitor treatment efficacy. Stiffness is emerging as a novel biomarker for assessing treatment efficacy in GBM; it can influence spheroid growth, invasion, and drug responsiveness [40]. It is known that healthy and pathologically altered tissues are different in their elasticity as well [41]. Viable tumor tissue and tumors experiencing cell necrosis have been associated with significant alterations in a tissue's viscoelastic properties due to a loss of tight junctions [27]. Therefore, there is potential for using stiffness for monitoring treatment efficacy, staging of cancer, and as a prognostic indicator [42], [43]. By these correlations, the treated LN229 spheroids very clearly displayed a marked decrease in size, cell viability, and stiffness over time when probed by both nb-OCE and Brillouin microscopy.

GBM, at its baseline, is highly resistant to treatment due to significant heterogeneity and its ability to activate pathways that promote cell survival and deactivate those that promote cell death [44]. Pertinently, the mechanical and chemical effects of specific cell types, LN229 and HA, have also been evaluated for their role in drug efficacy using the microwell platform [26], [45]. Astrocytes are responsible for promoting GBM growth and progression; communication between astrocytes and GBM through gap junction leads to increased intercellular calcium and resistance to chemotherapeutics [46]. The NF-κB signaling pathway regulates astrocyte formation in the GBM tumor microenvironment, and various studies have shown that NF-κB signaling in astrocytes can induce pro-inflammatory responses [47]. Reactive astrocytes, stimulated by gliomas, have also been known to increase the expression of MGMT (a DNA repair protein) to resist TMZ treatment [48]. The treated co-cultured LN229 and HA spheroids, when normalized, indicated that the tumor cells were experiencing decreased sensitivity to the treatment than when in monoculture. This is evident in smaller decreases in size, cell viability, and stiffness values over time versus the LN229 monoculture spheroids.

## V. Conclusion

In this study, we have provided multiple perspectives of the spheroids’ mechanical properties by joining the three experimental approaches of nb-OCE, Brillouin, and OCT. Nb-OCE and Brillouin microscopy were efficient and accurate methods for determining the stiffness of 400 μm spheroids, especially in combination with the assay platform. In our previous work, we investigated the efficacy of spheroid LN229 and HA co-culture model to mimic the tumor microenvironment and increase the sensitivity of GBM cells to chemotherapeutic treatment [26]. Here, we sought to confirm the correlation between tumor treatability, cell necrosis, and decreasing stiffness in the spheroids; stiffness was utilized as a biomarker for drug efficacy, of similar importance to cell viability. The monocultures and co-culture models from this study were found to yield results that are in agreement with the findings from our preceding work. Nb-OCE and Brillouin experiments revealed that the treated co-culture model demonstrated a smaller decrease in size and stiffness over time as compared to the treated GBM spheroids. This indicates a resistance to drug treatment, likely caused by the chemical and mechanical cues from the Human Astrocytes. Future studies should include other GBM cell lines, patient-derived cell lines, and more advanced drug combinations. This platform could also be applied to the investigation of the role of HUVEC cells in treatment efficacy and its relation to the enhanced permeability and retention (EPR) effect [49], as well as to the promotion of severe local invasion [50].

## Contributions

Megan Mendieta, Maryam Hatami, Yasemin Akay, Salavat Aglyamov, Kirill Larin, Konstantin Sokolov and Metin Akay designed the experiments. Megan Mendieta, Maryam Hatami, Sajedeh Saeidi, Mohammad Dehshiri, Dmitry Nevozhay, and Alexander Schill conducted the experiments. Megan Mendieta, Maryam Hatami, Sajedeh Saeidi, Manmohan Singh, Yasemin Akay, Kirill Larin, and Metin Akay analyzed and interpreted the data. Megan Mendieta, Maryam Hatami, Manmohan Singh, Dmitry Nevozhay, Alexander Schill, Salavat Aglyamov, Konstantin Sokolov, Yasemin M. Akay, Kirill Larin, and Metin Akay wrote and reviewed the manuscript.

## Supplementary Materials

Supplementary materials include a description and figures of the system setups for OCT, OCE, and Brillouin microscopy.

## Disclosures

KVL and MS have a financial interest in ElastEye LLC, which is not directly related to this work.
